# Female Sexual Dysfunction in Primary Adrenal Insufficiency

**DOI:** 10.3390/jcm10132767

**Published:** 2021-06-24

**Authors:** Virginia Zamponi, Pina Lardo, Roberta Maggio, Chiara Simonini, Rossella Mazzilli, Antongiulio Faggiano, Giuseppe Pugliese, Antonio Stigliano

**Affiliations:** Endocrinology Unit, Department of Clinical and Molecular Medicine, Sant’Andrea Hospital, Sapienza University of Rome, 00189 Rome, Italy; pina85la@gmail.com (P.L.); robertamaggio12@libero.it (R.M.); chiarasimonini594@gmail.com (C.S.); rossella.mazzilli@uniroma1.it (R.M.); antongiulio.faggiano@uniroma1.it (A.F.); giuseppe.pugliese@uniroma1.it (G.P.); antonio.stigliano@uniroma1.it (A.S.)

**Keywords:** adrenal insufficiency, Addison’s disease, female sexual dysfunction, cortisol, androgens, quality of life, sexuality, sexual medicine

## Abstract

Purpose. No data are currently available on female sexual dysfunction (FSD) in primary adrenal insufficiency (PAI) and the possible impact of replacement therapy. The aim of this study was to evaluate the prevalence of FSD and sexual distress (SD), and to evaluate the possible impact of replacement therapy on sexuality in women with PAI. Methods. Female Sexual Function Index-6 (FSFI-6) and Sexual Distress Scale (SDS) questionnaires were administered to 22 women with PAI and 23 healthy women matched for age as controls. Results. The prevalence of sexual symptoms measured by FSFI-6 (total score < 19) was significantly higher in women with PAI (15/22; 68.2%) compared to the controls (2/23; 8.7%; *p* = 0.001). Regarding the questionnaire items, significantly different scores were found for desire (*p* < 0.001), arousal (*p* = 0.0006), lubrication (*p* = 0.046) and overall sexual satisfaction (*p* < 0.0001) in women with PAI compared to the controls. The rate of FSD (FSFI < 19 with SDS >15) was 60% in patients with PAI. A significant inverse correlation was found between FSFI-6 total scores and SD (r = −0.65; *p* = 0.0011), while a significant direct correlation was found between FSFI-6 total scores and serum cortisol levels (r = 0.55; *p* = 0.035). Conclusions. A higher prevalence of FSD was found in women affected by PAI compared to healthy women. Desire seems to be the most impaired aspect of sexual function. Moreover, sexual dysfunction in this population seems to be related to sexual distress and cortisol levels.

## 1. Introduction

Primary adrenal insufficiency (PAI) is characterized by the destruction of the cortical portion of the adrenal gland with a depletion of the hormonal function, leading to a progressive deficiency in glucoactive, mineraloactive and androgen hormones. In Western countries, the main cause of Addison’s disease is an autoimmune reaction towards the adrenal cortex [1].

Nowadays, the treatment of PAI includes a replacement therapy with glucocorticoids (cortisone acetate, hydrocortisone) and mineralocorticoids (fludrocortisone) [2]. The lack of a therapeutic target marker often leads to the mentioned drugs being overdosed with a consequent loss of circadian rhythm. Both the hormonal deficit and the replacement therapy itself deeply impact the quality of life (QoL) of these patients [3]. Recently, pharmacological research has developed a modified-release hydrocortisone formulation in order to mimic the circadian rhythm of cortisol as closely as possible. Some studies report an improvement in QoL and metabolic parameters in patients treated with modified-release hydrocortisone [4,5,6]. Despite the optimization of this therapy, the patients affected by PAI consistently show reduced health-related QoL. Furthermore, the studies investigating QoL do not refer to the function and quality of sexual life.

It has been hypothesized that the lack of both glucocorticoids and androgens contributes to this condition; nonetheless, current evidence about the effects of dehydroepiandrosterone (DHEA) replacement therapy in women with androgen deficiency is still controversial. The latest guidelines of the Endocrine Society on PAI indicate the use of DHEA only in the presence of a decreased libido or depressed mood [7]. However, these two aspects are rarely explored in clinical settings.

Very few studies have evaluated gonadal, sexual and reproductive function in women with PAI, although a frequent association between PAI and primary ovarian failure is reported [8]. In the literature, only the study by Erichsen et al. has specifically investigated sexuality in women affected by PAI through the Sexual Activity Questionnaire (SAQ) [9]. It includes three sections that describe relationship status, reasons for sexual inactivity, and sexual functioning. The last section measures the pleasure and discomfort associated with sexual intercourse and changes according to the frequency of sexual activities. Unfortunately, the SAQ does not investigate sex-related personal distress or all the stages of female sexual response (desire, arousal, orgasm), and consequently does not allow for a definition of female sexual dysfunction (FSD) according to the criteria of Diagnostic and Statistical Manual of Mental Disorders, 5th Edition (DSM-5) [10]. Similar to hypogonadism in men, it is presumable that the lack of androgens in women suffering from PAI might negatively affect the phase of sexual desire. Moreover, few studies investigating female sexuality include personal sexual distress (SD); although in the DSM-5 [10], it has become a mandatory criterion for the diagnosis of FSD.

The aims of this study were to evaluate:

The prevalence of FSD in subjects affected by PAI compared to a healthy control group;

The correlation between sexual symptoms and SD; 

The impact of cortisol replacement therapy on sexual function in women with PAI.

## 2. Patients and Methods

In the present study, 45 women were enrolled from May 2019 to January 2020. Of these, 22 women were patients who attended the adrenal pathology outpatient clinic of our hospital and were affected by PAI (experimental group); 23 were healthy women without hormonal abnormalities or other comorbidities (control group) who were recruited from outpatients undergoing routine clinical screening for family history of thyroid goiter. 

The experimental group was composed of 2 patients with isolated PAI, 20 patients affected by type 2 autoimmune polyendocrine syndrome (APS2) including PAI and chronic thyroiditis with TSH falling within the normal range (mean = 2.7 ± 1.6 uIU/mL). All women with PAI were on replacement treatment; 12 of them were treated with hydrocortisone or cortisone acetate and 10 with a modified-release hydrocortisone formulation, considering 5.7–10 mg/m^2^ production rate. All women with PAI were also on replacement therapy with fludrocortisone, whose dosage was adjusted according to clinical (blood pressure) and biochemical (electrolytes and plasma renin activity) criteria.

Both patients with PAI and healthy controls met the following inclusion criteria: age 18–55 years; sexual activity over the last 4 weeks; heterosexual orientation; a stable relationship for at least one year; optimal replacement therapy with L-thyroxin for patients with APS2 suffering from thyroiditis with hypothyroidism; absence of thyroid nodules. 

The exclusion criteria were evidence of other ongoing acute or other chronic autoimmune or non-autoimmune pathologies; psychiatric diseases; primary ovarian insufficiency; taking psychiatric drugs or estrogen/progesteron replacement therapy; evidence of sexual disorders in the male partners; APS2 with Type 1 Diabetes Mellitus (T1DM). 

All subjects enrolled in the present study underwent a detailed medical history collection and a physical examination including a Body Mass Index (BMI kg/m^2^) calculation. Both the patient and control groups were administered the FSFI-6 and the SDS questionnaires. 

In addition, in patients affected by PAI, the following aspects were considered: (a) cortisol and ACTH concentrations: blood for the determination level of both was collected in the morning at 8.00 A.M. Plasma ACTH and serum cortisol were assayed by the ELISA method (Cusabio–Houston, TX, USA); (b) treatment of PAI (hydrocortisone or cortisone acetate/modified-release hydrocortisone formulation); (c) AddiQoL score. 

The study was conducted according to the guidelines of the hospital’s ethics committee. Written informed consent was obtained from all participants included in the study.

### 2.1. Questionnaires

FSFI-6 is a validated questionnaire that allows the identification of sexual problems. The original version, FSFI-19 [11], includes 6 domains that investigate sexual symptoms, according to the International Classification of Diseases-10 (ICD-10) [12] and the DSM-5 [10]: desire, arousal, lubrication, orgasm, satisfaction and dyspareunia. Compared to the extended version, the FSFI-6 allows rapid screening to identify FSDs [13]. Isidori et al. indicated a cut-off of 19 for FSFI-6, which demonstrates a high sensitivity, specificity and positive and negative predictive values for the identification of women with positive screening for FSD in a sample of Caucasian women [14].

Sexually related personal distress is mandatory for the diagnosis of FSD [10]. SDS was developed, validated and is among the most widely used tools to measure distress associated with impaired sexual disfunction, demonstrating strong internal consistency, reliability, and validity [15]. SDS includes 13 items referring to different negative emotional responses related to sex: including worry, anxiety, frustration, bother, and feelings of inadequacy. The importance of considering SD is underscored by its inclusion as a criterion for the diagnosis of sexual dysfunctions in the DSM-5 [10]. Scores > 15 are compatible with sex-related stress. 

AddiQoL is a validated questionnaire consisting of 30 items that specifically investigates QoL in patients with PAI [16]. It has high internal consistency and reliability and its validity as a QoL instrument in PAI was further substantiated by its high correlation with SF-36, a generic QoL questionnaire that is widely used and thoroughly validated [17]. 

### 2.2. Statistical Analysis

Mean ± standard deviation were calculated for all measured variables. The D’Agostino Pearson Normality Test was used to assess the normality of distributions. Unpaired t tests and Mann–Whitney tests were used to detect statistical differences between the FSFI-scores of the experimental and control groups. The differences in FSD prevalence were assessed (referring to a total FSFI-6 score ≤ 19) by Fisher’s exact test. Finally, Pearson and Spearman correlation tests were carried out between the total scores of FSFI-6 questionnaires and SDS and ACTH and cortisol levels of patients, as appropriate. A *p*-value of 0.05 was considered for the statistical procedures. Statistical analysis was carried out with GraphPad Prism software (Version 7.0, GraphPad Software Inc., San Diego, CA, USA).

## 3. Results

### 3.1. Patients

The study and control populations were matched for the main characteristics. No individual consumed more than three standard alcoholic drinks per week (Table 1). The mean ± SD age of the experimental group was 46.6 ± 5.8; the mean ± SD age of control the group was 47.3 ± 4.8. The mean ± SD BMI of the experimental group was 23.8 ± 4.6; the mean ± SD BMI of control the group was 21.4 ± 3.4. No significant differences regarding the mean age (*p* = 0.09) and BMI (*p* = 0.07) were found between the experimental and control groups.

### 3.2. FSFI-6 Questionnaire

**Figure 1:**
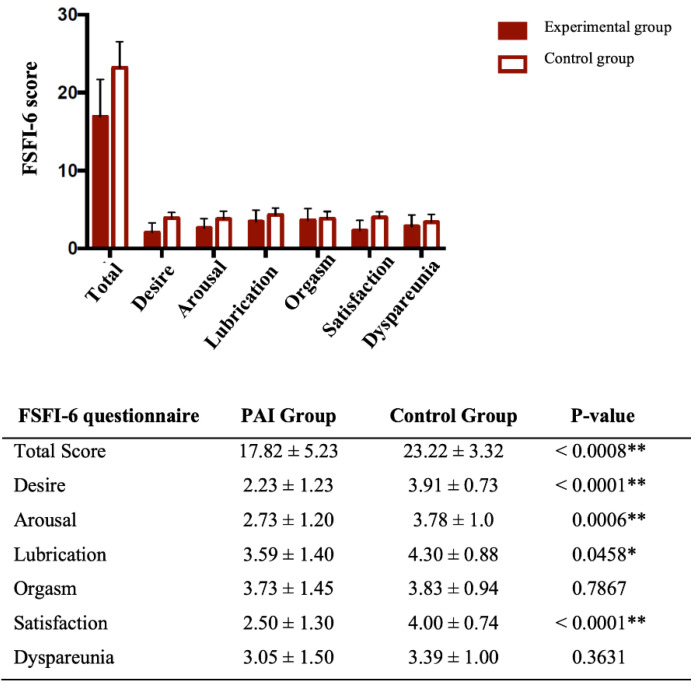
The graph bars represent total score and single items (mean ± SD) of FSFI-6 in the primary adrenal insufficiency (PAI) group and the control group. In the table below, the same value of total score and single items (mean ± SD) of patients and controls are displayed together with *p* values (Unpaired *t* tests, Mann–Whitney tests) (** *p* < 0.001; * *p* < 0.01).

The prevalence of sexual symptoms (total score ≤ 19) was significantly higher in the patients’ group (15/22, 68.2%) compared to the control group (2/23, 8.7% *p* < 0.0001). The relative risk (RR) was 7.84 with a 95% IC of 2.41–28.84. Total scores were significantly lower in the PAI group compared to the control group (*p* < 0.0008). Regarding the single items of the questionnaire, a significant difference was found for desire, arousal, lubrication and sexual satisfaction (*p* < 0.0001, *p* = 0.0006, *p* = 0.0458 and *p* < 0.0001, respectively). The results are summarized in Figure 1.

**Figure 2:**
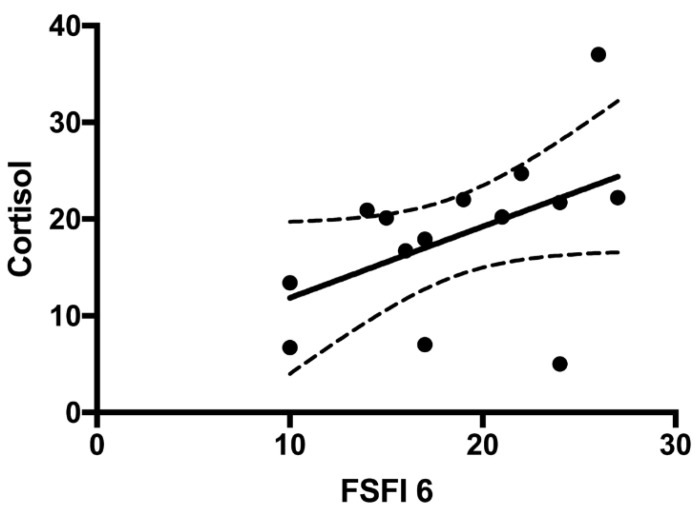
Correlation between FSFI-6 total score and serum cortisol levels in patients’ group (Spearman correlation test; *p* = 0.03; r = 0.55).

The prevalence of sexual problems was 80% (8/10) in the group of women treated with a modified-release hydrocortisone formulation compared to 58.3% (7/12) in the group of women treated with hydrocortisone or cortisone acetate (*p* = 0.38). A comparison between women treated with hydrocortisone or cortisone acetate and those treated with the modified-release hydrocortisone formulation showed no significant differences between any of the FSFI-6 items or their total scores. On the contrary, a significant direct correlation was found between FSFI-6 total scores and cortisol levels (r = 0.55; *p* = 0.035) (Figure 2).

No statistically significant correlations were found between ACTH plasma levels and total scores for the FSFI-6 questionnaire (*p* = 0.2 r = −0.3) (data not shown).

### 3.3. Sexual Distress Questionnaire (SDS)

**Figure 3:**
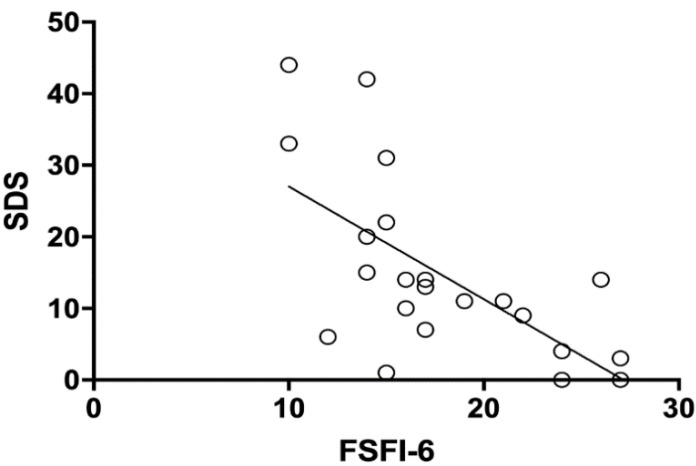
Correlation between FSFI-6 total scores and sexual distress in adrenal insufficiency group (Pearson correlation test; *p* = 0.001; r-0.65).

The prevalence of SD (total score > 15) was significantly higher in the PAI (10/22, 45.5%) compared to the control group (2/23, 8.7% *p* = 0.007). The relative risk (RR) was 5.22, with a 95% IC of 1.51–19.90. The prevalence of SD in patients affected by PAI with a of FSFI score < 19 was 60% (9/15). A significant inverse correlation was found between SDS total scores and FSFI-6 total scores (Pearson correlation analysis r = −0.6482, IC 95%: −0.8402 to −0.3118; *p* = 0.0011) (Figure 3). 

### 3.4. AddiQol

Mean ± SD AddiQoL score of the PAI group was 96.6 ± 13.7. No statistically significant correlations were found between AddiQoL and FSFI-6 total score questionnaire (*p* = 0.68 r = 0.1) (data not shown). No statistically significant correlations were found between the item relating to sexuality in the AddiQoL and FSFI-6 total score questionnaire (*p* = 0.2 r = 0.3) (data not shown).

## 4. Discussion

In the present study, sexual response and sex-related personal distress in women with PAI were investigated. Sexuality plays an important role in a woman’s quality of life; it is influenced by several biological, psychological and socio-relational factors [18]. Sexual function still remains an unexplored field for clinicians, as its evaluation can be influenced by several variables. The only and most promising tool at the moment is represented by validated items. First of all, the choice of an appropriate questionnaire is a pivotal factor, as it should specifically take into account the sexual response according with DSM-5 [10]. In order to formulate a diagnosis of FSD, by definition, a clinically significant distress associated with sexual symptoms should be present in at least 75% of sexual experiences [10]. Interestingly, most of the available data in literature cannot provide a real FSD diagnosis, because distress associated with sexual symptoms is rarely measured. In a large-scale investigation on American healthy women, the authors attempted to assess both the prevalence of sexual symptoms and the rate of associated distress. The prevalence of sexual symptoms was 44.2%, whereas sexually related personal distress was observed in only 22.8% of respondents. Overall, these results indicate that FSD may be overestimated when SD is not considered [19]. In our study, a statistically significant higher rate of sexual symptoms measured by FSFI-6 was found in women with PAI compared to healthy controls (*p* = 0.001). The total FSFI-6 score and the subdomains of desire, arousal, lubrication and satisfaction were significantly lower in patients suffering from PAI (*p* < 0.0001, *p* = 0.0006, *p* = 0.0458 and *p* < 0.0001, respectively). In our study population, desire was the phase of sexual response that appeared to be most impaired compared to controls (Figure 1). Moreover, a significant difference in SD prevalence between PAI and the control group was found (*p* = 0.007) and the prevalence of FSD (women with sexual symptoms and related SD) remained high (60%). Furthermore, a significant inverse correlation was found between FSFI-6 total scores and SDS total scores (r = −0.6482, *p* = 0.0011), showing a concordance between these two parameters. 

It could be hypothesized that the higher prevalence of FSD in the PAI group is due to an androgen deficiency, as it also happens to men affected by hypogonadism. Dehydroepiandrosterone sulfate (DHEA-S) is the major androgen of the adrenal cortex, an inactive precursor that is metabolized into active androgens and/or estrogens in specific peripheral tissues. There is some evidence that a reduction in DHEA-S levels could lead to impaired quality of life, low libido and lack of well-being [20]. In this regard, several studies investigated female sexual function in other androgen deficiency conditions. Nevertheless, these studies showed contradictory results. In 2016, the Fourth International Consultation of Sexual Medicine highlighted the important role of androgens in female sexual function and the efficacy of androgen replacement therapy for the treatment of some women with hypoactive sexual desire disorder (HSDD) [21]. Concerning the role of androgens in PAI, the available data are still under debate. In 1999, Arlt et al. [22] demonstrated that women with PAI experienced improved libido as well as mood and well-being when treated with a DHEA replacement therapy [23,24]. On the other hand, many authors report that females with PAI do not show reduced sexual activity despite subnormal levels of androgens. For example, Hunt et al. [23] found no effect on sexual function in women with PAI treated with an equivalent dose of DHEA. Nonetheless, these authors reported a beneficial relative effect in relation to the overall well-being and reduced fatigue. Other studies reported similar results, not showing any positive impact of a DHEA replacement therapy on the sexual function of women with PAI [25,26,27]. Currently, only Erichsen et al. have tried to investigate sexuality in women with Addison’s disease through SAQ [9]. The authors did not report a significant statistic difference in sexual activity between PAI patients using DHEA than patients without androgen replacement therapy. Moreover, no significant correlation between androgen metabolites and sexual activity and pleasure was found. In the same paper, women with PAI in comparison with women underwent to salpingo-oophorectomy with normal adrenal function, showed a sexual pleasure reduction [9], suggesting that other adrenal hormones besides androgens are involved in female sexual function. In addition to androgen deficiency, an underestimated or overestimated glucoactive replacement therapy could be at the basis of sexual dysfunction. Nonetheless, to our knowledge no data exist in literature regarding a possible association between FSD and glucocorticoids replacement therapy. Tops et al. [28] reported that high levels of cortisol lead to an increase in oxytocin levels. Oxytocin typically increases during sexual intercourse and is thought to play a key role in sexual arousal and orgasm [29,30,31,32]. In a small study of 12 men with autoimmune PAI, Granata et al. reported an association with many sexual dysfunctions, with a significant improvement of some parameters after cortisol replacement therapy. The change in erectile function was positively correlated with the cortisol levels and renin activity variation, suggesting that cortisol plays a role in erectile dysfunction in patients with PAI [33]. In contrast, regarding sexual function in women with Cushing Syndrome [34], some authors found a higher prevalence of FSD in women suffering from endogenous hypercortisolism compared to controls indicating that an excess of hormone level can have a negative impact of female sexual function. In the present study, a significant direct correlation between FSFI-6 total scores and serum cortisol levels after replacement therapy (r = 0.55; *p* = 0.035) was found. No significant differences between women treated with hydrocortisone or cortisone acetate and with the modified-release hydrocortisone formulation for any of the FSFI-6 items or total score were seen. Several studies report an improvement in QoL in patients treated with modified-release hydrocortisone, which maintains cortisol levels in a more physiological range [4,5,6]. Taking into account this data, it could be hypothesized that an appropriate cortisol replacement dose together with the respect of the circadian rhythm can play a significant role not only on QoL but also on female sexual function. In the present study, no correlation was found between FSD and QoL. Moreover, no correlation was found between the FSFI-6 total score and the only item concerning sexuality included in the AddiQoL questionnaire. These data confirm that specific questionnaires about sexual aspects are needed to identify FSD.

The present study has some limitations that must be considered when interpreting the results. Only heterosexual women were included in the present study. Furthermore, the small number of the sample may reduce the statistical significance of the obtained results, especially concerning the impact of different glucoactive therapies on sexuality in women with PAI, which should be considered. Clinical trials on large scale are needed to better investigate the role of glucoactive hormones in female sexual function. 

## 5. Conclusions

Women affected by PAI have a higher risk of developing FSD than healthy women of the same age. The prevalence of sexual symptoms remains high in this population even considering the personal distress which may positively correlate with the severity of sexual symptoms. Hypoactive sexual desire disorder appears to be the most represented sexual dysfunction in patients with PAI. The inverse correlation between FSD and cortisol levels could suggest a key role of glucocorticoid deficiency and replacement therapy in sexual function in women with PAI.

## Figures and Tables

**Table 1:** Demographic features of primary adrenal insufficiency (PAI) group and control group.

	PAI Group	Control Group	*p*
Age (years; mean ± SD)	46.6 ± 5.8	47.3 ± 4.8	0.09
Higher education level (University, Specialization, Master, PhD)	n. 15/22 (68.2%)	n. 17/23 (73.9%)	0.75
Smoking habits	n. 3/22 (13.6%)	n. 5/23 (21.7%)	0.69
Occasional alcohol consumption	n. 2/22 (9%)	n. 6/23 (26.0%)	0.24
Menopause	n. 10/22 (45.4%)	n. 9/23 (39.1%)	0.77
Parity	n. 14/22 (63.6%)	n. 12/23 (52.2%)	0.55
BMI (kg/m^2^; mean ± SD)	23.8 ± 4.6	21.4 ± 3.4	0.07

## Data Availability

The data that support the findings of this study are available from the corresponding author, V.Z. upon reasonable request.

## Abbreviations

FSD: Female Sexual Dysfunction; PAI: Primary Adrenal Insufficiency; SD: Sexual Distress; FSFI-6: Female Sexual Function Index-6; SDS: Sexual Distress Scale; QoL: Quality of Life; DHEA: Dehydroepiandrosterone; SAQ: Sexual Activity Questionnaire; DSM-5: Diagnostic and Statistical Manual of Mental Disorders, 5th Edition; APS2: Autoimmune Polyendocrine Syndrome type 2; T1DM: Type 1 Diabetes Mellitus; BMI: Body Mass Index; ICD-10: International Classification of Diseases-10; DHEA-S: Dheydroepiandrosterone Sulfate; HSDD: Hypoactive Sexual Desire Disorder.
